# Parenclitic network mapping predicts survival in critically ill patients with sepsis

**DOI:** 10.14814/phy2.70407

**Published:** 2025-06-06

**Authors:** Emily Ito, Tope Oyelade, Matthew Wikner, Jinyuan Liu, Watjana Lilaonitkul, Ali R. Mani

**Affiliations:** ^1^ Network Physiology Lab, Division of Medicine UCL London UK; ^2^ Institute of Health Informatics, UCL London UK; ^3^ Department of Perioperative Medicine and Pain Barts Health NHS Trust London UK; ^4^ Global Business School for Health, UCL London UK; ^5^ Institute for Liver and Digestive Health (ILDH), Division of Medicine UCL London UK

**Keywords:** intensive care, network, network physiology, parenclitic, sepsis, survival

## Abstract

Sepsis is a complex disease involving multiple organ systems. A network physiology approach to sepsis may reveal collective system behaviors and intrinsic organ interactions. However, mapping functional connectivity for individual patients has been challenging due to the lack of analytical methods for evaluating physiological networks using routine clinical and laboratory data. This study explored the use of parenclitic network mapping to assess organ connectivity and predict sepsis outcomes based on routine laboratory data. Data from 162 sepsis patients meeting Sepsis‐3 criteria were retrospectively analyzed from the MIMIC‐III database. Fifteen physiological variables representing organ systems were used to construct organ network connectivity through correlation analysis. Correlation analysis identified 7 interactions linked to 30‐day survival. Parenclitic network analysis was used to measure deviations in individual patients' correlations between organ systems from the reference physiological interactions observed in survivors. Parenclitic deviations in the pH‐bicarbonate axis (hazard ratio = 2.081, *p* < 0.001) and pH‐lactate axis (hazard ratio = 2.773, *p* = 0.024) significantly predicted 30‐day mortality, independent of the Sequential Organ Failure Assessment (SOFA) score and ventilation status. This study highlights the potential of parenclitic network mapping to provide insights into sepsis pathophysiology and differences in organ system connectivity between survivors and non‐survivors independent of sepsis severity and mechanical ventilation status.

## 1 INTRODUCTION

Sepsis is a complex life‐threatening disorder due to dysregulated host response to systemic infection that leads to multiorgan failure (Singer et al., 2016). The global burden of sepsis is substantial, with around 48.9 million estimated worldwide cases and 11 million deaths annually (Fleischmann et al., 2016; Rudd et al., 2020). The body's response to systemic infection in sepsis is highly *dynamic*. Immune homeostasis, which is essential for orchestrating effective response against infection, is dysregulated in sepsis, causing haemodynamic and metabolic changes that lead to organ dysfunction (Salomão et al., 2019). While improved intensive care unit (ICU) management has increased survival rates in the early hyperinflammatory phase of sepsis, the persistence of the disease can result in a shift from immune resistance to immune tolerance (Delano & Ward, 2016). Patients who develop immunosuppression suffer from long‐term mortality due to impaired capacity to eradicate both the primary pathogen and nosocomial infections (Denstaedt et al., 2018). This makes sepsis a complex disease as both hyperinflammation and immunosuppression play an important role in the progression of sepsis and its survival (Hotchkiss et al., 2013). Physiological organs' response to systemic inflammation is diverse and depends on metabolic state and energetic trade‐offs, which eventually may lead to deterioration, organ dysfunction, or disease tolerance (Ganeshan et al., 2019). Moreover, factors such as age, infection origin, and comorbidities also influence the sepsis trajectory, contributing to disease heterogeneity (Denstaedt et al., 2018).

**FIGURE 1:**
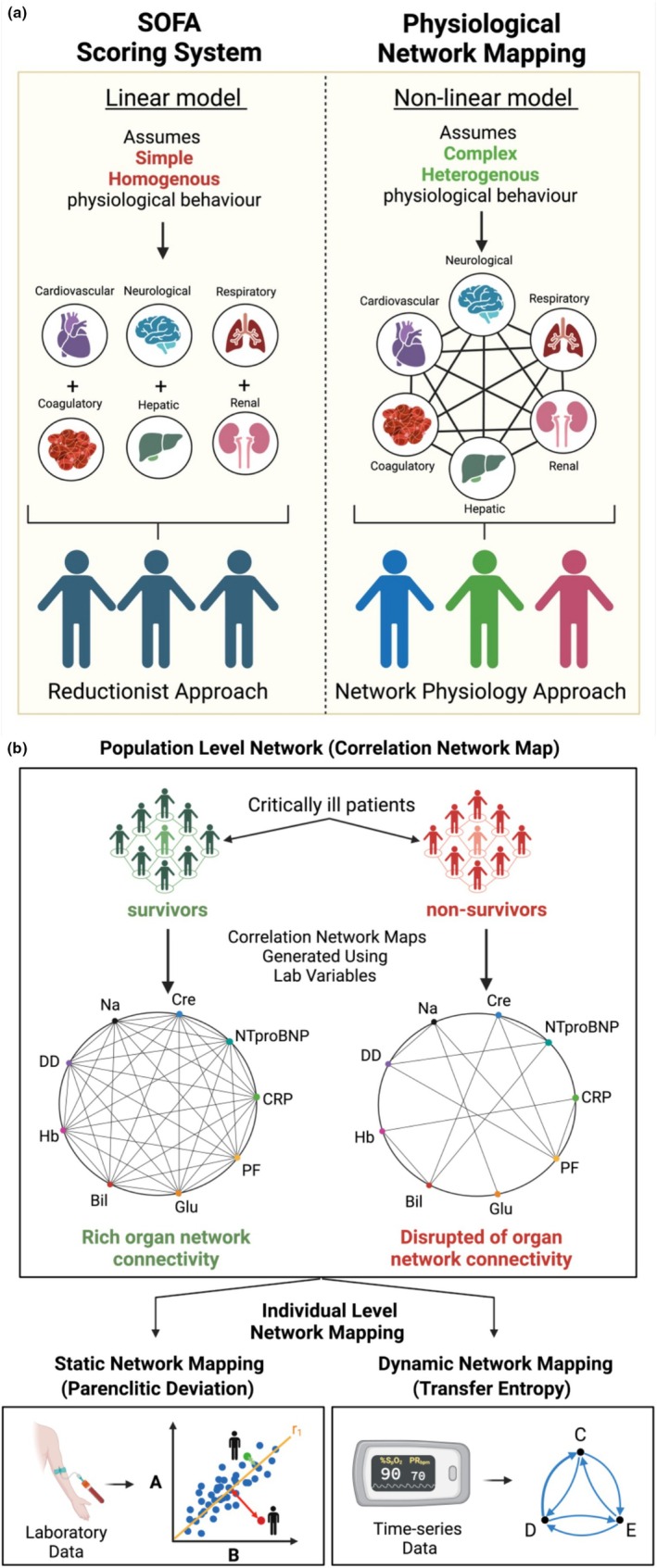
(a) Diagram illustrating the differences between linear and non‐linear models of sepsis pathophysiology. (b) Diagram illustrating different network physiological techniques used for population‐level (A) and individual‐level (B) analysis of organ network connectivity.

Over the past decades, numerous attempts have been made to develop effective treatments, but most clinical trials have failed due to challenges posed by the complex pathophysiology of sepsis and patient heterogeneity (Marshall, 2014). There is strong evidence suggesting that early identification and treatment of at‐risk sepsis populations (e.g., early identification of deterioration) improve patient outcomes (Seymour et al., 2017). Current methods for evaluating sepsis, like Sequential Organ Failure Assessment (SOFA), are based on linear models of sepsis pathophysiology, where the severity of organ dysfunction is estimated by treating each organ as an independent entity and summing the level of dysfunction across all organs (Figure 1a) (Vincent et al., 1996). However, these methods have a limited ability to identify early sepsis, potentially due to the simplistic nature of the linear models, which can overlook the complex interplay between organ systems that is generated in response to systemic infection during sepsis (Godin & Buchman, 1996; Moorman et al., 2016; Quinten et al., 2018).

Network physiology is an emerging field that studies complex interactions within the physiological system (Bashan et al., 2012). Unlike SOFA, which takes a linear approach, the network physiology approach considers the interaction between physiological components, making it suitable for investigating physiological behaviors in complex diseases like sepsis (Figure 1a) (Asada et al., 2016; Godin & Buchman, 1996).

Numerous studies have highlighted the critical role of organ system connectivity in health and disease (Asada et al., 2016; Bhogal & Mani, 2017; Jiang et al., 2021; Morandotti et al., 2025; Oyelade et al., 2023; Zhang et al., 2022). A pioneering report by Asada et al. investigating organ network connectivity in critically ill patients has yielded some promising findings (Asada et al., 2016). The study examined the physiological network in patients by selecting physiological variables from the blood test that represent different organ systems and analyzing the correlation amongst these variables. They showed that in critically ill patients with matched severity of organ dysfunction (i.e., SOFA), non‐survivors had much less significant correlations between physiological variables compared to survivors. These findings lead to two important conclusions: (1) organ network disruption is associated with poor patient outcomes, and (2) studying organ interactions may yield independent information regarding patient outcomes, in addition to linear models such as SOFA. There is growing evidence showing that sepsis is associated with the uncoupling of physiological compensatory mechanisms and reduced organ system engagement (Gheorghita et al., 2022; Gholami et al., 2012; Godin & Buchman, 1996; Papaioannou et al., 2012). Therefore, studying organ connectivity in sepsis might yield important information about patient outcomes.

The correlation network mapping approach introduced by Asada et al. is an effective technique for analyzing physiological network interactions, but it is limited to population‐level analysis. In contrast, techniques such as parenclitic deviation (PD) and transfer entropy (TE) investigate organ network connectivity in individual patients using static (i.e., laboratory variables) and dynamic data (i.e., physiological time‐series such as heart rate), respectively (Figure 1b) (Schreiber, 2000; Zanin et al., 2014). PD evaluates physiological interactions by looking at how individual patient data deviate from the reference population characteristics (i.e., in survivors) (Zhang et al., 2022). Meanwhile, TE measures organ connectivity by computing the bidirectional information transfer between physiological systems (Schreiber, 2000).

A recent report by Morandotti et al. (2025) employed TE analysis to study the cardio‐respiratory network in septic patients using publicly available data from the MIMIC‐III database (Johnson et al., 2016; Morandotti et al., 2025). The study demonstrated that the evaluation of organ connectivity in sepsis patients using TE could predict 30‐day survival and 48‐hour deterioration, independent of SOFA and ventilation status. While TE shows potential as a novel tool for detecting early sepsis deterioration, its clinical application may be challenging due to the need for noise‐free time‐series data, which can be hard to obtain in intensive care units, particularly in resource‐limited settings (Berntson & Stowell, 1998; Lee & Jung, 2018).

One major advantage of PD is that it can be computed using a single measurement from a routine laboratory test (i.e., routine blood test), requiring minimal patient involvement. Parenclitic analysis has demonstrated positive results in predicting disease outcomes for numerous conditions, such as acute liver failure, cirrhosis, and ovarian and prostate cancer (Morandotti et al., 2025; Oyelade et al., 2024; Whitwell et al., 2018; Zhang et al., 2022). However, network mapping with PD has never been applied in sepsis research.

This research builds on the previous work by Morandotti et al. (2025) and investigates the same sepsis population from the MIMIC‐III database. The primary aim of this study was to investigate whether evaluation of organ network connectivity through the parenclitic analysis of routinely collected physiological data can predict 48‐h deterioration and 30‐day mortality in sepsis patients.

## 2 MATERIALS AND METHODS

**FIGURE 2:**
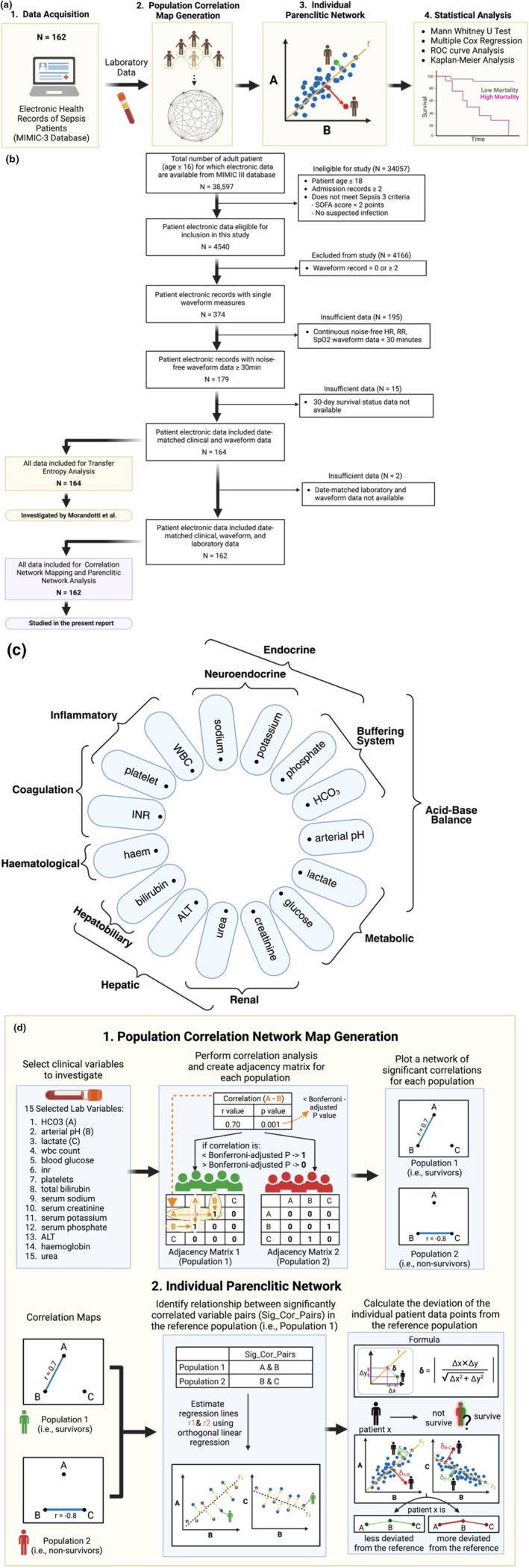
(a) An overview of the research design. (b) The flowchart illustrates the complete data extraction process from the MIMIC‐III database. The purple boxes show the patient data included in the present work, investigating the application of static network mapping (parenclitic analysis) in predicting sepsis outcomes. The yellow boxes highlight the study population included in the earlier work by Morandotti et al. (2025), which explored the use of dynamic network mapping (transfer entropy) in prognosticating sepsis outcomes. (c) The figure illustrates 15 physiological variables representing different organ systems. Each variable is categorized according to the systems that it represents. Variables belonging to more than one category may reflect the function of multiple physiological systems. (d) Process behind parenclitic network mapping. (1) Population correlation map generation. (2) Computation of individual parenclitic network for significant physiological correlations identified from population correlation maps. “*r*” represents the correlation coefficient for the regression line of significantly correlated physiological variables (i.e., A and B) within the reference population (i.e., survivors). “” represents the parenclitic deviation (PD). “∆” represents the difference between the reference population and individual patient data. All PD computations were conducted using in‐house code developed in MATLAB.

This retrospective cohort study was performed using electronic medical records from critically ill sepsis patients admitted to Beth Israel Deaconess Medical Center in Boston, Massachusetts, obtained through the Medical Information Mart for Intensive Care (MIMIC‐III) database (Johnson et al., 2016). An overview of the study design is illustrated in Figure 2a.

### 2.1 Ethics statement

Patient information from the MIMIC‐III database was deidentified in compliance with Health Insurance Portability and Accountability Act (HIPAA) standards. The MIMIC project was approved by the Institutional Review Boards of Beth Israel Deaconess Medical Center and the MIT (IRB protocol nos. 2001P001699/14 and 0403000206, respectively). Prior to the study, the authors involved in data processing completed a mandatory online ethics training at MIT and were accredited (IDs: 10304625 and 48067739).

### 2.2 Patient data extraction

Structure query language (SQL) code was used to extract patient data from the MIMIC‐III database. A flowchart detailing the complete data extraction process is shown in Figure 2b.

The inclusion criteria were patients above 18 years old diagnosed with sepsis who had a single record of ICU admission. An SQL script developed by Johnson et al. was used to identify sepsis cases based on the Sepsis 3 criteria: an acute increase in SOFA score ≥2 points and suspicion of infection (Johnson et al., 2018; Seymour et al., 2016; Singer et al., 2016).

This paper is a part of a larger study investigating the role of physiological network mapping in sepsis. A recent study by Morandotti et al. (2025) evaluated dynamic network mapping in sepsis using physiological time‐series data. In contrast, the present work examines parenclitic network mapping using point‐in‐time physiological data. We used the same patient population (*N* = 164) analyzed by Morandotti et al. (2025) to allow comparison between different network mapping techniques. These patients were initially selected based on the availability of date‐matched clinical and waveform data (Figure 2b). For a detailed description of the sampling criteria, please refer to Morandotti et al. (2025).

In the present study, laboratory data (LABEVENTS) were extracted for each patient (*N* = 164) using their unique patient identifier code (SUBJECT_ID). Only patients with the laboratory data corresponding to the same ICU stay as the waveform and clinical data were included. Date‐matched laboratory data were missing for two patients; hence, the final cohort for the correlation network mapping and parenclitic analysis comprised 162 patients.

### 2.3 Selection of the clinical and physiological variables

The clinical data extracted include the age, sex, ethnicity, SOFA score, Elixhauser Comorbidity Index, ventilation status, 30‐day survival follow‐up, 30‐day survival status, and 48‐h deterioration outcome. The SOFA score was calculated on two occasions: first, on the earliest day when both the laboratory and waveform data were available, and second, after 48 h. Deterioration was defined as an increase in SOFA by at least 2 points at 48 h. The details of SOFA calculation and the definition of deterioration are described elsewhere (Morandotti et al., 2025).

For the laboratory data, 15 physiological variables representing different organ systems were retrieved, including serum phosphate, arterial pH, urea, hemoglobin (haem), lactate, white blood cell count (WBC), serum sodium, international normalized ratio (INR), platelets, total bilirubin, blood glucose, serum creatinine, alanine transaminase (ALT), bicarbonate (HCO_3_^−^), and serum potassium. These selected variables represent key physiological systems involved in maintaining body homeostasis, which are commonly affected during sepsis: inflammatory (Foy et al., 2022), coagulation (Levi & Meijers, 2011; Schupp et al., 2022), hematological (Goyette et al., 2004; Peng et al., 2024), hepatic (Ferrario et al., 2021; Kubilay et al., 2016; Lin et al., 2022), renal (Han et al., 2021), metabolic (Fitzgerald et al., 2023; Liu et al., 2022), acid–base homeostasis (Achanti & Szerlip, 2023; Guyton & Hall, 2006; Seifter, 2014), and endocrine systems (Centeno et al., 2019; Dawson, 1984) (Figure 2c).

The mean level of variables measured within the first 24 h of ICU admission was used for the parenclitic analysis. All laboratory data come from routine blood tests. The arterial pH was measured directly using an arterial blood gas (ABG) analyzer, and the HCO_3_^−^ levels were obtained separately from the basic metabolic panel. The pH and the bicarbonate levels were not estimated using the Henderson‐Hasselbalch equation to ensure that the correlation observed between pH and HCO_3_^−^ is purely physiological and not due to the mathematical relationship.

### 2.4 Correlation network map generation in a sepsis population

Correlation network maps were generated to investigate organ connectivity at the population level (Figure 2d). 162 patients were separated according to their 30‐day survival and 48‐h deterioration outcome. Correlations between 15 physiological variables were analyzed as described by Asada et al. (2016). Pearson's correlation analysis was conducted with a pair‐matching technique to account for the missing data. Patient data were omitted from the analysis whenever either of the two variables being investigated was missing (Tan et al., 2020). Correlation between two variables was considered significant if the significance level was less than the Bonferroni‐adjusted *p*‐value (*p* ≤ 0.00047619). The results from the correlation analysis were used to form an adjacency matrix, where “0” indicates a lack of significant correlation and “1” represents a significant correlation between two physiological variables (Figure 2d). Correlation networks were visualized for 30‐day survival and 48‐h deterioration using the adjacency matrix to plot a graph. Nodes represent the physiological variables, and edges show a significant correlation between the two variables. The edge thickness was weighted according to the strength of Pearson's correlation coefficient of individual correlations to illustrate the relative strength of each correlation within the network. All codes for generating the correlation network map were developed in MATLAB build R2024a (MATLAB, 2024).

### 2.5 Parenclitic network mapping of individual patients

Significant correlations identified from the population correlation network maps for 30‐day survival and 48‐h deterioration were used to calculate parenclitic deviations (PD). Parenclitic analysis was conducted for individual patients (*N* = 162) by investigating how the relationship between physiological variables in each patient deviated from the general trend in the reference population, which consisted of the survivor cohort for 30‐day survival and the non‐deteriorated patient group for 48‐h deterioration (Oyelade et al., 2023; Zhang et al., 2022). For each pair of significant correlations between the physiological variables, its general trend in the reference population was identified by performing a regression analysis (Figure 2d). The orthogonal linear regression (OLS) was chosen over the classical least squares (CLS) method for the regression line estimation because OLS accounts for errors in the measurement of both dependent and independent variables, unlike CLS, which assumes that measurement errors only occur in the dependent variable (Keleş, 2018). Following the estimation of the regression line representing the trend in the reference population, PD was computed by measuring the orthogonal distance between each patient's datapoint and the regression line using the equation shown in Figure 2d (Zhang et al., 2022). All the PDs were computed using an algorithm built in MATLAB build R2024a (MATLAB, 2024). The code developed by the author is publicly available on GitHub: https://github.com/emilyito/Parenclitic‐Deviation‐OLR.

### 2.6 Statistical analysis

All the statistical analysis was conducted using SPSS Statistics 29 (Corp., I. B. M, 2023). Data are reported as mean ± standard deviation and the significance level was set at 0.05 for statistical tests unless stated otherwise. Mann–Whitney *U* test was performed to compare differences in PDs and SOFA scores between survivors and non‐survivors for 30‐day survival and deteriorated and non‐deteriorated patients for 48‐hour deterioration. Multivariate Cox regression was performed to investigate whether the computed PD values significantly predict 30‐day survival and 48‐h deterioration, independent of SOFA score and ventilation status. Since PDs look at the relationship between two variables (i.e., PD (pH‐lactate)) and each variable could independently influence the disease outcome, the mean values of the analyzed physiological variables (i.e., such as pH and lactate for PD (pH‐lactate)) were also included as covariates. PD values used in the multivariate Cox regression were normalized using Z‐transformation prior to analysis. Receiver Operating Characteristic (ROC) curve analysis was performed for PD axes that significantly predicted 30‐day survival. Area Under the Curve (AUC) from the ROC curve analysis was obtained to assess and compare the diagnostic performance between PDs and SOFA. Delong's test was conducted using MedCalc Statistical Software version 23.2. to compare the ROC curves (DeLong et al., 1988; MedCalc Software Ltd., 2025). Youden index from the ROC curve analysis was used as a cut‐off with the optimum balance of sensitivity and specificity for classifying patients into predicted survivor and non‐survivor groups. Sepsis patients classified into predicted survivor and non‐survivor groups were used to generate the Kaplan–Meier Curve for 30‐day survival. The Log‐Rank test was used to compare the survival times between the two groups.

## 3 RESULTS

### 3.1 General characteristics of the study population

Out of 162 patients included in the study, 30 patients deteriorated at the 48‐h follow‐up, and 33 patients passed away within 30 days of ICU admission. Sepsis patients with poor disease outcomes, including non‐survivors and deteriorated patients, were older, had a higher SOFA score, and had a greater likelihood of having a positive ventilation status compared to survivors and non‐deteriorated patients (Table 1). The number of patients with positive ventilation status was significantly different for both 30‐day survival and 48‐h deterioration (*p* < 0.001). Significant differences in age (*p* = 0.008) and SOFA score (*p* < 0.001) were only found for 30‐day survival. For the physiological variables, phosphate, urea, lactate, and creatinine levels were significantly elevated, and bicarbonate and arterial pH were significantly reduced in non‐survivors and deteriorated patients compared to survivors and non‐deteriorated patients. The white blood cell count (*p* < 0.045) and glucose level (*p* < 0.019) were only significantly elevated in non‐survivors for 30‐day survival. Conversely, alanine transaminase (*p* < 0.043) was only significantly elevated in deteriorated patients for 48‐h deterioration.

**TABLE 1:** Demographic and clinical characteristics of the study population with different 30‐day survival and 48‐h deterioration outcomes.

General characteristics of survivors and non‐survivors			
	Survivors (*n* = 129)	Non‐survivors (*n* = 33)	*p*‐Value
Age	66.3 (51.5–82.3)	76.1 (67.6–86.1)	**0.008**
Male sex, *n* (%)	71 (55%)	23 (69.7%)	0.128
Ethnicity, *n* (%)			
White	101 (78.3%)	22 (66.7%)	0.163
Black	7 (5.4%)	3 (9.1%)	0.435
Hispanic	3 (2.3%)	2 (6.1%)	0.268
Asian	3 (2.3%)	0 (0%)	0.377
Other	3	0	0.377
Unknown	12	6	0.148
SOFA score	4 (2–5)	6 (3–10)	**<0.001**
Elixhauser Index	22 (19–25)	24 (19–27.5)	0.078
Positive ventilation status, *n* (%)	27 (20.9%)	18 (54.5%)	**<0.001**
Positive 48 h deterioration status, *n* (%)	8 (6.2%)	22 (66.7%)	**<0.001**
*Physiological variables*			
Phosphate, mg/dL	3.1 (2.4–3.7)	3.9 (2.8–4.4)	**0.004**
Arterial pH	7.39 (7.36–7.43)	7.32 (7.20–7.42)	**0.024**
Urea, mg/dL	16.5 (10.5–23.3)	23.3 (15–35.9)	**0.003**
Hemoglobin, mg/dL	11.2 (9.9–12.4)	11.7 (10.4–12.5)	0.269
Lactate, mmol/L	1.6 (1.2–2.4)	3.8 (1.3–5.9)	**0.002**
White blood cell count, ×1000/μL	10.8 (8.3–14.1)	13 (9.8–16.2)	**0.045**
Sodium, mEq/L	139 (136.8–141)	139 (136.1–143)	0.738
INR	1.2 (1.1–1.4)	1.3 (1.2–1.8)	**0.004**
Platelet count, ×1000/μL	217 (149–267.5)	187.3 (138–286.5)	0.49
Bilirubin, mg/dL	0.6 (0.4–1.6)	0.68 (0.43–1.15)	0.76
Glucose, mg/dL	124.5 (109.2–150.8)	143.6 (119.1–178.6)	**0.019**
Creatinine, mg/dL	0.9 (0.7–1.2)	1.1 (0.9–1.7)	**0.004**
Alanine Transaminase, U/L	28 (17–59.5)	30 (16.8–149.1)	0.653
Bicarbonate, mEq/L	24 (22–26)	22 (16.6–26.8)	**0.042**
Potassium, mEq/L	4.2 (3.6–4.5)	3.9 (3.4–5)	0.967

General characteristics of deteriorated and non‐deteriorated patients			
	No deterioration (*n* = 132)	Deterioration (*n* = 30)	*p* Value
Age	67.4 (52.1–84.5)	72.6 (61.8–82.4)	0.211
Male sex, *n* (%)	75 (56.8%)	19 (63.3%)	0.514
Ethnicity, *n* (%)			
White	101 (76.5%)	22 (73.3%)	0.713
Black	9 (6.8%)	1 (3.3%)	0.474
Hispanic	4 (3%)	1 (3.3%)	0.931
Asian	3 (2.3%)	0 (0%)	0.405
Other	2 (1.5%)	1 (3.3%)	0.505
Unknown	13 (9.8%)	5 (16.7%)	0.283
SOFA score	4 (2–6)	4.5 (3–9.3)	0.089
Elixhauser Index	22 (19–26)	22 (19–26)	0.867
Positive ventilation status, *n* (%)	30 (22.7%)	15 (50%)	**0.003**
Positive 30‐day mortality, *n* (%)	11 (8.3%)	22 (73.3%)	**<0.001**
*Physiological variables*			
Phosphate, mg/dL	3.2 (2.4–6.7)	3.8 (2.6–4.4)	**0.023**
Arterial pH	7.39 (7.36–7.44)	7.32 (7.19–7.39)	**<0.001**
Urea, mg/dL	16 (10.5–24)	22.2 (15.5–31.8)	**0.005**
Hemoglobin, mg/dL	11.2 (9.9–12.3)	11.7 (10.6–12.7)	0.158
Lactate, mmol/L	1.6 (1.1–2.4)	3.7 (2.2–6)	**<0.001**
White blood cell count, ×1000/μL	10.9 (8.3–14.1)	11.7 (9.7–16.4)	0.236
Sodium, mEq/L	139 (137–141)	139.5 (135.6–143)	0.645
INR	1.2 (1.1–1.4)	1.3 (1.2–1.5)	0.145
Platelet count, ×1000/μL	217 (147–274)	187.3 (148.5–282.8)	0.803
Bilirubin, mg/dL	0.6 (0.4–1.6)	0.68 (0.48–0.9)	0.939
Glucose, mg/dL	126 (109.8–151.5)	146.7 (115.9–160.8)	0.106
Creatinine, mg/dL	0.9 (0.75–1.2)	1.1 (0.84–1.7)	**0.028**
Alanine Transaminase, U/L	26 (16–53)	52.8 (24.1–162.8)	**0.043**
Bicarbonate, mEq/L	24 (22–26)	22 (16–25.3)	**0.032**
Potassium, mEq/L	4.2 (3.6–4.4)	3.9 (3.3–4.9)	0.865

*Note*: Data is shown as median (lower quartile – upper quartile) unless stated otherwise as frequency (*n*) and percentage (%). The chi‐squared test was used to evaluate differences in the distribution of positive ventilation, deterioration, mortality, male sex, and ethnicity. Differences in the distribution of other variables were assessed using the Mann–Whitney *U* test. Significant differences in the studied variables (*p* < 0.05) are highlighted in bold.

### 3.2 Population correlation network maps identify distinct correlation profiles for 30‐day survival and 48‐h deterioration

**FIGURE 3:**
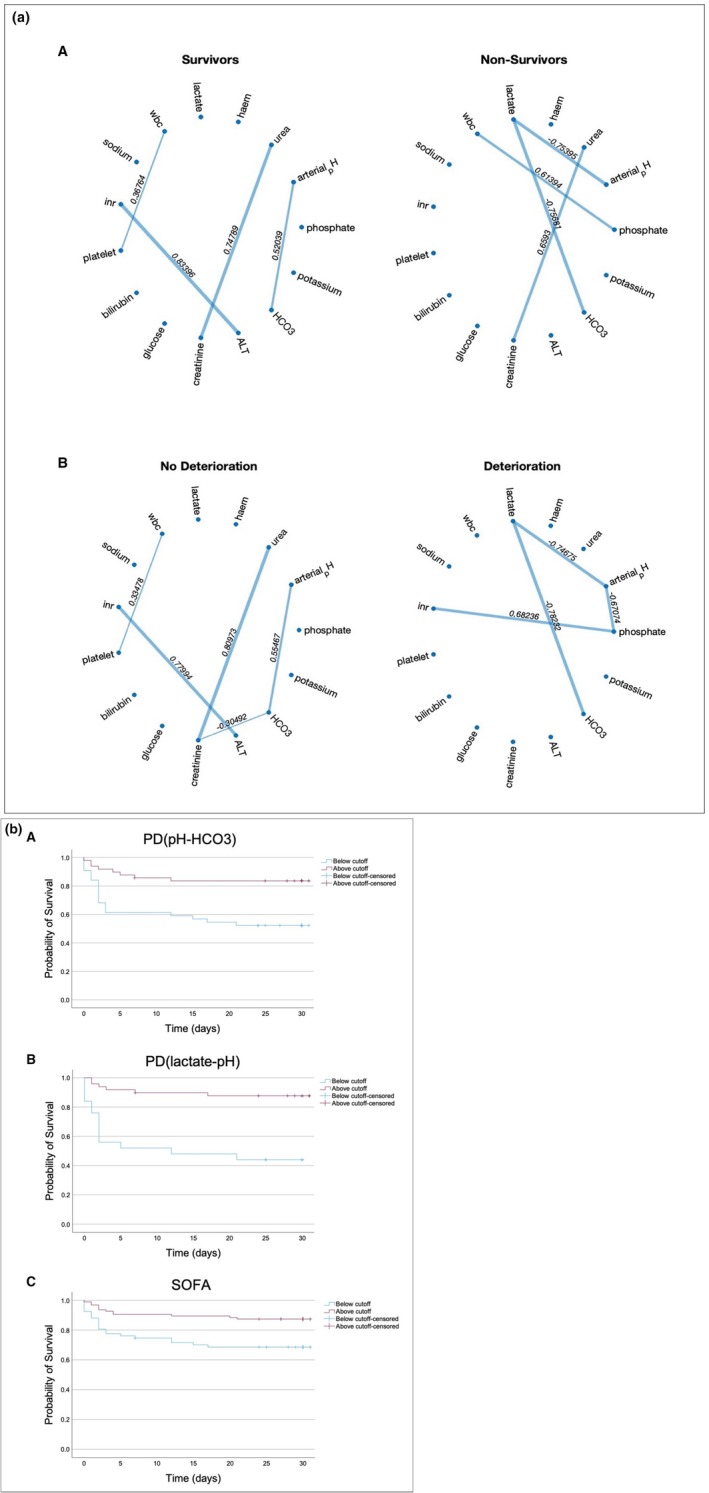
(a) Population Correlation Network Maps for 30‐day survival (A) and 48‐h deterioration (B). Nodes represent physiological variables. Edges show significant correlations (*p*‐value ≤Bonferroni‐adjusted *p*‐value) between the two nodes. Edge labels display the values of Pearson's correlation coefficient. (b) Kaplan–Meier Graphs illustrating 30‐day survival of patients classified into predicted survivor and non‐survivor groups based on (A) PD (pH – HCO_3_^−^), (B) PD (lactate – pH), and (C) SOFA score cut‐offs.

Correlation Network Maps were generated for 162 patients, classified according to 30‐day survival and 48‐h deterioration, to explore the population‐level physiological network interactions (Figure 3a). Four pairs of significantly correlated variables were identified in both survivors and non‐survivors for 30‐day survival (Bonferroni‐corrected *p*‐value ≤0.00047619). Similarly, for 48‐h deterioration, four and five pairs of significant physiological correlations were found in non‐deteriorated patients and deteriorated patients, respectively. Significant physiological correlations were unique between survivors and non‐survivors for 30‐day survival and between non‐deteriorated and deteriorated patients for 48‐h deterioration, except for the correlation between urea and creatinine, which was significant in both survivors and non‐survivors. Overall, the correlation profile differed between patients with positive and negative disease outcomes (i.e., survivors vs. non‐survivors and non‐deteriorated vs. deteriorated patients); however, it was similar within groups of patients with positive outcomes (i.e., survivors versus non‐deteriorated patients) and negative outcomes (i.e., non‐survivors versus deteriorated patients) (Figure 3a).

### 3.3 Parenclitic deviations of significant physiological correlations predict 30‐day mortality independent of SOFA score and ventilation status, but not 48‐h deterioration in critically ill sepsis patients

Parenclitic deviations (PD) were calculated individually for 162 patients across all significant physiological correlations identified from the population correlation network maps. PDs across the correlated variables were generally higher for non‐survivors and deteriorated patients compared to survivors and non‐deteriorated patients with small to moderate effect size (*r*) (Tables 2 and 3) (Cohen, 1988).

**TABLE 2:** Mann–Whitney *U* test results looking at the differences in parenclitic deviations (PD) between survivors “0” and non‐survivors “1” for 30‐day survival.

	30‐day survival	*N*	Mean	SD	Mann–Whitney *U* test		
					*U*	*r*	*p*‐Value
*Significant pairs in survivors*							
PD (WBC – platelets)	0	127	3.151	3.096	2164	0.068	0.392
	1	31	4.861	6.199			
PD (INR – ALT)	0	54	0.207	0.251	613	0.147	0.209
	1	19	0.350	0.385			
PD (pH – HCO_3_^−^)	0	64	0.045	0.034	1342	0.356	**<0.001**
	1	29	0.095	0.076			
PD (urea – creatinine)	0	127	0.219	0.174	2409	0.128	0.105
	1	32	0.416	0.506			
*Significant pairs in non‐survivors*							
PD (WBC – phosphate)	0	119	0.658	0.454	2328	0.183	**0.025**
	1	31	1.204	1.438			
PD (lactate – pH)	0	53	0.045	0.038	844.5	0.455	**<0.001**
	1	20	0.163	0.127			
PD (lactate – HCO_3_^−^)	0	60	0.813	0.855	927.5	0.461	**<0.001**
	1	19	2.391	1.978			
PD (urea – creatinine)	0	127	0.219	0.174	2409	0.128	0.105
	1	32	0.416	0.506			

*Note*: PD axes significantly different between the two groups are highlighted in bold (*p* < 0.05).

**TABLE 3:** Mann–Whitney *U* test results looking at the differences in parenchymal deviations (PD) between non‐deteriorated “0” and deteriorated “1” patients for 48‐h deterioration.

PD axis	48‐h deterioration	*N*	Mean	SD	Mann–Whitney *U* test		
					*U*	*r*	*p*‐Value
*Significant pairs in non‐deteriorated patients*							
PD (WBC – platelets)	0	129	3.140	2.804	2001	0.047	0.558
	1	29	5.130	6.825			
PD (INR – ALT)	0	56	0.248	0.293	523	0.072	0.540
	1	17	0.276	0.245			
PD (pH – HCO_3_^−^)	0	68	0.046	0.037	1242	0.352	**<0.001**
	1	25	0.098	0.079			
PD (creatinine – HCO_3_^−^)	0	131	0.320	0.309	2585.5	0.240	**0.002**
	1	29	0.554	0.532			
PD (urea – creatinine)	0	131	0.213	0.173	2341.5	0.182	**0.022**
	1	28	0.462	0.538			
*Significant pairs in deteriorated patients*							
PD (lactate – pH)	0	54	0.052	0.042	678	0.243	**0.038**
	1	19	0.141	0.130			
PD (lactate – HCO_3_^−^)	0	61	0.839	0.853	817	0.352	**0.002**
	1	18	2.364	2.027			
PD (phosphate – pH)	0	64	0.055	0.046	982.5	0.177	0.096
	1	25	0.104	0.098			
PD (phosphate – INR)	0	105	0.272	0.417	1239	0.064	0.467
	1	26	0.187	0.149			

*Note*: The two groups with significantly different (*p* < 0.05) PD values are highlighted in bold.

Results from the Mann–Whitney *U* test revealed significant differences in PD between survivors and non‐survivors for 30‐day survival along the pH‐HCO_3_^−^ axis (*p* < 0.001), WBC‐phosphate axis (*p* = 0.025), lactate‐pH axis (*p* < 0.001), and lactate‐HCO_3_^−^ axis (*p* < 0.001) (Table 2). Similarly, for 48‐h deterioration, significant differences in PD between patients who did not deteriorate and those who did deteriorate were found along the pH‐HCO_3_^−^ axis (*p* < 0.001), creatinine‐HCO_3_^−^ axis (*p* = 0.002), urea‐creatinine axis (*p* = 0.022), lactate‐pH axis (*p* = 0.038), and lactate‐HCO_3_^−^ axis (*p* = 0.002) (Table 3).

Subsequently, multivariate Cox regression analysis showed that PDs along pH‐HCO_3_^−^ (hazard ratio, 95% CI = 2.081, 1.361–3.184) and lactate‐pH (hazard ratio, 95% CI = 2.773, 1.143–6.731) axes significantly predict 30‐day mortality independent of SOFA and ventilation status (Table 4). These results suggest that one standard deviation increase in PD (pH‐HCO_3_^−^) and PD (lactate‐pH) can increase the risk of 30‐day sepsis mortality by approximately twofold (95% CI = 36% – 3 folds) and threefold (95% CI = 14% – 6 folds), respectively. The ability of PD (pH‐HCO_3_^−^) and PD (lactate‐pH) to predict 30‐day mortality remained statistically significant (*p* < 0.01) after adjusting for additional covariates, including age, gender, ethnicity, and Elixhauser comorbidity index (Table S1). None of the PDs computed for significant correlation in deteriorated and non‐deteriorated patients predicted 48‐h deterioration independent of SOFA and ventilation status (Table 5).

**TABLE 4:** Results from multivariate Cox regression evaluating whether parenclitic deviations (PD) can predict 30‐day sepsis survival, independent of SOFA and ventilation status.

Covariates	β	SE	Hazard ratio	95% CI (Hazard ratio)		*p*‐Value
				Lower	Upper	
*Significant pairs in survivors*						
PD (WBC – platelets)	−0.152	0.272	0.859	0.504	1.463	0.575
WBC	0.493	0.304	1.638	0.903	2.969	0.104
Platelets	−0.055	0.207	0.947	0.630	1.422	0.791
SOFA	0.215	0.054	1.240	1.115	1.378	**<0.001**
Ventilation Status	1.214	0.364	3.367	1.651	6.867	**<0.001**
PD (INR – ALT)	−0.142	0.421	0.868	0.380	1.981	0.737
INR	0.665	0.557	1.945	0.653	5.792	0.232
ALT	−0.801	0.850	0.449	0.085	2.378	0.346
SOFA	0.116	0.068	1.123	0.982	1.283	0.090
Ventilation Status	1.414	0.498	4.114	1.551	10.913	**0.004**
PD (pH – HCO_3_^−^)	0.733	0.217	2.081	1.361	3.184	**<0.001**
Arterial_pH	−0.147	0.252	0.864	0.527	1.416	0.561
HCO_3_^−^	−0.160	0.205	0.852	0.571	1.273	0.435
SOFA	0.059	0.061	1.061	0.941	1.196	0.334
Ventilation Status	0.830	0.387	2.293	1.074	4.893	**0.032**
PD (urea – creatinine)	0.193	0.207	1.213	0.808	1.821	0.352
Urea	0.272	0.181	1.312	0.921	1.870	0.133
Creatinine	−0.059	0.313	0.943	0.510	1.743	0.851
SOFA	0.137	0.068	1.146	1.004	1.309	**0.043**
Ventilation Status	1.533	0.395	4.634	2.137	10.048	**<0.001**
*Significant pairs in non‐survivors*						
PD (WBC – phosphate)	−0.057	0.273	0.945	0.553	1.614	0.836
WBC	0.257	0.163	1.293	0.939	1.781	0.116
Phosphate	0.428	0.299	1.534	0.853	2.757	0.153
SOFA	0.155	0.053	1.168	1.053	1.296	**0.003**
Ventilation Status	1.229	0.372	3.418	1.648	7.089	**<0001**
PD (lactate – pH)	1.020	0.452	2.773	1.143	6.731	**0.024**
Lactate	0.137	0.275	1.147	0.668	1.968	0.619
Arterial_pH	−0.042	0.359	0.959	0.474	1.939	0.908
SOFA	0.060	0.075	1.062	0.916	1.231	0.428
Ventilation Status	0.514	0.471	1.671	0.664	4.207	0.275
PD (lactate – HCO_3_^−^)	0.811	0.585	2.249	0.714	7.086	0.166
Lactate	−0.349	0.594	0.706	0.220	2.259	0.557
HCO_3_^−^	−0.245	0.198	0.782	0.530	1.154	0.216
SOFA	0.108	0.076	1.114	0.959	1.294	0.158
Ventilation Status	0.985	0.510	2.678	0.985	7.276	0.053
PD (urea – creatinine)	0.193	0.207	1.213	0.808	1.821	0.352
Urea	0.272	0.181	1.312	0.921	1.870	0.133
Creatinine	−0.059	0.313	0.943	0.510	1.743	0.851
SOFA	0.137	0.068	1.146	1.004	1.309	**0.043**
Ventilation Status	1.533	0.395	4.634	2.137	10.048	**<0.001**

*Note*: The values of PD in this table were all normalized by z‐transformation prior to the analysis. Significant predictors of 30‐day mortality (*p* < 0.05) are highlighted in bold.

**TABLE 5:** Multivariate Cox regression analysis investigating whether parenclitic deviations (PD) can predict 48‐h sepsis deterioration, independent of SOFA and ventilation status.

Covariates	β	SE	Hazard ratio	95% CI (Hazard ratio)		
				Lower	Upper	*p*‐Value
*Significant pairs in non‐deteriorated patients*						
PD (WBC – platelets)	0.025	0.246	1.025	0.633	1.662	0.919
WBC	0.233	0.274	1.262	0.737	2.161	0.397
Platelets	−0.077	0.211	0.926	0.612	1.400	0.715
SOFA	0.078	0.058	1.082	0.966	1.211	0.172
Ventilation Status	0.959	0.373	2.609	1.256	5.418	**0.010**
PD (INR – ALT)	−0.222	0.406	0.801	0.362	1.773	0.584
INR	0.365	0.511	1.441	0.530	3.919	0.474
ALT	−0.493	0.688	0.611	0.159	2.353	0.474
SOFA	0.090	0.071	1.094	0.952	1.257	0.204
Ventilation Status	0.718	0.491	2.051	0.783	5.369	0.144
PD (pH – HCO_3_^−^)	0.255	0.256	1.291	0.781	2.134	0.319
Arterial_pH	−0.326	0.355	0.722	0.360	1.447	0.358
HCO_3_^−^	−0.087	0.237	0.917	0.576	1.459	0.714
SOFA	−0.028	0.074	0.972	0.842	1.123	0.700
Ventilation Status	0.595	0.412	1.813	0.808	4.064	0.149
PD (creatinine – HCO_3_^−^)	0.143	0.256	1.153	0.698	1.904	0.577
Creatinine	0.046	0.285	1.047	0.598	1.831	0.873
HCO_3_^−^	−0.207	0.191	0.813	0.558	1.183	0.279
SOFA	0.022	0.072	1.022	0.887	1.177	0.765
Ventilation Status	0.922	0.380	2.515	1.196	5.293	**0.015**
PD (urea – creatinine)	0.312	0.210	1.366	0.906	2.060	0.137
Urea	0.277	0.242	1.320	0.821	2.121	0.252
Creatinine	−0.230	0.335	0.795	0.412	1.531	0.492
SOFA	0.039	0.073	1.039	0.900	1.200	0.600
Ventilation Status	1.104	0.410	3.016	1.351	6.737	**0.007**
*Significant pairs in deteriorated patients*						
PD (lactate – pH)	−0.626	0.615	0.535	0.160	1.784	0.308
Lactate	0.584	0.320	1.793	0.958	3.355	0.068
Arterial_pH	−0.911	0.620	0.402	0.119	1.355	0.141
SOFA	−0.016	0.081	0.984	0.839	1.155	0.845
Ventilation Status	0.646	0.474	1.908	0.753	4.837	0.173
PD (lactate – HCO_3_^−^)	0.088	0.605	1.093	0.334	3.575	0.884
Lactate	0.393	0.674	1.482	0.395	5.554	0.560
HCO_3_^−^	−0.073	0.204	0.930	0.624	1.386	0.720
SOFA	0.013	0.083	1.013	0.861	1.191	0.879
Ventilation Status	0.787	0.501	2.198	0.823	5.868	0.116
PD (phosphate – pH)	−0.327	0.426	0.721	0.313	1.663	0.443
Phosphate	0.052	0.153	1.054	0.782	1.421	0.731
Arterial_pH	−0.906	0.523	0.404	0.145	1.127	0.083
SOFA	−0.024	0.072	0.976	0.848	1.124	0.739
Ventilation Status	0.496	0.409	1.642	0.737	3.657	0.225
PD (phosphate – INR)	−0.554	0.438	0.575	0.244	1.355	0.206
Phosphate	0.231	0.164	1.260	0.914	1.737	0.159
INR	0.205	0.392	1.228	0.569	2.648	0.601
SOFA	0.050	0.069	1.051	0.918	1.202	0.470
Ventilation Status	1.035	0.414	2.816	1.250	6.344	**0.012**

*Note*: All the PD values in this table were z‐normalized prior to the analysis. Significant predictors of 48‐h deterioration (*p* < 0.05) are highlighted in bold.

### 3.4 Cut‐offs for Parenclitic deviations Across pH‐HCO_3_ ^−^ and lactate‐pH axes significantly predict 30‐Day sepsis mortality

ROC curve analysis was conducted to evaluate the diagnostic performance of PD(pH‐HCO_3_^−^), PD(lactate‐pH) and SOFA score in classifying sepsis patients based on their 30‐day survival (*p* ≤ 0.001). The results indicated that all three measures were moderate classifiers of 30‐day sepsis survival. However, the AUC for PDs along pH‐HCO_3_^−^ (AUC, 95% CI = 0.723, 0.607–0.839) and lactate‐pH (AUC, 95% CI = 0.797, 0.667–0.927) axes showed a slightly better performance compared to SOFA (AUC, 95% CI = 0.708, 0.602–0.814) with increases in AUC by 2% and 12%, respectively (Table 6). Although the AUCs for PD(pH‐HCO_3_^−^) and PD(lactate‐pH) were higher than that of SOFA, the differences were not statistically significant (Tables S2 and S3).

**TABLE 6:** Results from ROC Curve Analysis of parenclitic deviations significantly predictive of 30‐day mortality (PDs for pH‐HCO_3_^−^ and lactate‐pH axes) and SOFA (*p* < 0.05).

Variables	AUC	95% CI for AUC		*p* Value	Youden index	Sensitivity (%)	Specificity (%)
		Lower	Upper				
PD (pH – HCO_3_^−^)	0.723	0.607	0.839	0.001	0.051	72.4	64.1
PD (lactate – pH)	0.797	0.667	0.927	0.000	0.071	70	79.2
SOFA	0.708	0.602	0.814	0.000	4.5	63.6	64.3

*Note*: Sensitivity and specificity were calculated at the cut‐off point identified by the Youden Index. Each variable was analyzed individually using available data: PD (pH‐HCO_3_^−^) (*N* = 93), PD (lactate‐pH) (*N* = 73), and SOFA (*N* = 162).

Abbreviations: AUC, Area under the Curve; CI, Confidence Interval.

The Youden Index identified from ROC analysis was used as cut‐offs for classifying patients according to their predicted survival status (Table 6). Kaplan–Meier graphs were plotted for PD(pH‐HCO_3_^−^), PD(lactate‐pH) and SOFA to compare 30‐day survival between the predicted survivors and non‐survivors (Figure 3b). Log‐rank test showed that survival rates were significantly lower in patients classified beyond the cut‐offs compared to below the cut‐offs for PD(pH‐HCO_3_^−^) (52.3% vs. 83.7%, *p* < 0.001), PD(lactate‐pH) (44.0% vs. 87.8%, *p* = 0.001) and SOFA (68.7% vs. 87.4%, *p* = 0.002). The differences in survival rates between patients beyond and below the cut‐offs were larger for PDs along pH‐HCO_3_^−^ (∆ = 31.5%) and lactate‐pH (∆ = 43.8%) axes compared to SOFA (∆ = 18.7%) (Figure 3b).

## 4 DISCUSSION

This report is the first study to investigate physiological network interactions in a sepsis cohort using parenclitic network analysis. 162 critically ill sepsis patients were studied retrospectively using health record data from the MIMIC‐III database. Fifteen laboratory variables representing different physiological systems were selected to study organ network connectivity in our study population. The main goal of this study was to evaluate whether parenclitic network analysis can predict early sepsis outcomes, independent of other markers of sepsis severity. The results showed that PDs for the pH‐bicarbonate and pH‐lactate axes significantly predict 30‐day mortality in sepsis patients independent of SOFA score, ventilation status, Elixhauser Comorbidity Index, age, gender, and ethnicity. None of the PDs were able to predict 48‐h deterioration.

### 4.1 Correlation map showed a distinct pattern of physiological network interactions between sepsis patients with different disease outcomes

The population correlation analysis was conducted to investigate the pattern of organ network connectivity in patients with different 48‐h deterioration and 30‐day survival outcomes. Network correlation maps have shown significant differences in correlation profiles between patients with positive and negative disease outcomes (i.e., survivors versus non‐survivors). However, the amount of significant physiological correlations (edges) was similar between survivors and non‐survivors and between non‐deteriorated and deteriorated patients. This indicates that despite the change in the correlation pattern, there was no absolute loss of network connectivity in patients with negative disease outcomes. These findings were different from previous studies investigating organ connectivity in critically ill patients and with patients with chronic liver conditions. A study by Asada et al. investigating critically ill patients from the ICU over a period of 6 months found that non‐survivors had considerably fewer significant physiological correlations compared to survivors (Asada et al., 2016). Similarly, other studies looking at patients with cirrhosis and chronic liver failure showed that patients who did not survive had a more disrupted organ network (less numbers of edges) compared to those who survived after 3‐, 6‐, and 12‐month follow‐ups (Oyelade et al., 2023; Tan et al., 2020; Zhang et al., 2022).

However, a recent study investigating patients with paracetamol‐induced acute liver failure (ALF) reported findings that aligned with our results (Oyelade et al., 2024). Population correlation network analysis on ALF patients showed that while there was a general decrease in organ connectivity in non‐survivors than in the survivors after 28‐day follow‐up, the scale of disruption was much less severe than in other studies investigating organ connectivity in patients with chronic liver disease (Oyelade et al., 2023, 2024; Tan et al., 2020; Zhang et al., 2022). Similarly to our findings, the study showed that the distinct pattern of organ connectivity between survivors and non‐survivors was more evident than the presence of physiological network disruption.

A possible explanation for these alignments and discrepancies between the referenced studies and our findings may be due to the variations in the duration of patient follow‐up and differences in the nature of the diseases being investigated (i.e., acute vs. chronic). Like the study on acute liver failure, which reported results aligning with ours, our study investigated short‐term patient mortality by following up the study population for only a month. In contrast, the other studies reporting organ network disruption have investigated patients with chronic conditions (i.e., chronic liver failure) and followed them for a more extended period (3–12 months).

Sepsis is a complex condition, with patients suffering from both short‐term and long‐term disease outcomes (Delano & Ward, 2016). The absence of a disrupted network may be due to differences in physiological response toward acute and chronic conditions. Acute physiological changes in sepsis are often associated with the rearrangement of the organ network as the body attempts to resist and eliminate infectious pathogens (i.e., via coordination of pro and anti‐inflammatory responses and metabolic reprogramming) (Oyelade et al., 2024; Willmann & Moita, 2024). Conversely, chronic changes may exhibit a disrupted organ network connectivity due to the development of tolerance (i.e., immunosuppression and chronic inflammation) (Delano & Ward, 2016; Oyelade et al., 2024). Since our study has followed up on the patients for only a month, those who passed away likely did so during the acute stage of disease progression due to organ failure from a maladaptive early response in an attempt to resolve the infection. This likely led to a distinct pattern of organ connectivity in sepsis non‐survivors, rather than a disruption.

### 4.2 Acid–Base compensatory mechanism and its important role in sepsis survival

Following the population correlation map generation, we investigated organ network connectivity in individual sepsis patients using parenclitic network analysis. Our findings demonstrated that PDs across significant physiological correlations were generally greater in non‐survivors and deteriorated patients compared to survivors and non‐deteriorated patients, indicating that organ connectivity was more disrupted in patients with negative disease outcomes while it remained intact in those with positive outcomes. Interestingly, PD values were significantly different between patients with positive and negative disease outcomes across physiological correlations related to metabolic system and acid–base homeostasis, like pH‐HCO_3_^−^, lactate‐pH, lactate‐HCO_3_^−^ for both 30‐day survival and 48‐h deterioration, and creatinine‐HCO_3_^−^ for 48‐h deterioration only (Tables 2 and 3). While none of the computed PDs predicted 48‐h deterioration, results from Cox regression analysis showed that PDs across the pH‐HCO_3_^−^ and lactate‐pH axes were significantly predictive of 30‐day sepsis survival independent of other markers of sepsis severity. This suggests that effective regulation of the acid–base compensatory mechanism could play an important role in sepsis survival.

Cellular metabolism is a widely investigated topic in sepsis research, and metabolic dysfunction in sepsis patients has been reported for almost a century (Liu et al., 2022; Thomson, 1929). There is an early shift from oxidative phosphorylation (aerobic respiration) to glycolysis (anaerobic respiration) in immune cells, allowing rapid energy production for their activation, proliferation, and differentiation, all of which are essential for generating an effective response to infection during sepsis (Liu et al., 2022). However, excessive glycolytic activity and failure to restore oxidative phosphorylation in sepsis have been associated with the accumulation of lactate (a by‐product of anaerobic respiration) and reduced blood pH (Suetrong & Walley, 2016). Under normal physiological conditions, a decrease in blood pH is countered by bicarbonate buffering, returning pH to its physiological range. However, in sepsis, persistent lactatemia due to excessive glycolysis can overwhelm the bicarbonate buffer system and deplete the plasma bicarbonate levels (Kamel et al., 2020). This dysregulation of the acid–base homeostasis can lead to metabolic acidosis and contribute to organ dysfunction.

The underlying mechanisms behind dysfunction in cellular metabolism and the acid–base compensatory system in sepsis remain an active area of research. Numerous studies investigating skeletal muscle and liver samples in sepsis have shown that reduced mitochondrial activity is linked to poor disease outcomes (Brealey et al., 2002; Carré et al., 2010; Haden et al., 2007). In particular, nitric oxide (NO) is overproduced in sepsis due to inflammatory stress and is known to affect mitochondrial respiration (Brown, 1999; Cauwels, 2007). An animal study investigating the effects of NO on mitochondrial activity has shown that high levels of NO can inhibit mitochondrial complexes I, II, III, and IV by competing with oxygen, thereby reducing aerobic respiration. While the inhibition of complex II, III, and IV was reversible, prolonged exposure to high levels of NO irreversibly inhibited complex I and led to cytotoxic effects (Clementi et al., 1998). Other studies investigating muscle biopsies from septic patients also demonstrated significantly elevated NO production, reduced mitochondrial complex I activity, lower ATP production, and elevated serum lactate levels in non‐survivors compared to survivors (Brealey et al., 2002; Carré et al., 2010). These results support that persistent NO inhibition of mitochondrial enzymes can result in permanent mitochondrial damage. Additionally, they provide strong evidence that the inability to restore oxidative metabolism may lead to a cascade of events (i.e., lactate overproduction due to increased anaerobic respiration, metabolic acidosis, and organ dysfunction), ultimately resulting in poor sepsis outcomes (Suetrong & Walley, 2016). Consequently, the increase in PD along the pH‐lactate axis in non‐survivors may be due to physiological uncoupling in the metabolic system, particularly in the homeostasis between glycolysis and oxidative phosphorylation.

Nonetheless, metabolic dysfunction and lactate overproduction are not the only factors that may contribute to dysregulated acid–base balance in sepsis non‐survivors. Other processes, such as reduced bicarbonate production, are also likely involved. Under healthy conditions, the loss of endogenous bicarbonate is primarily compensated by the liver via the Cori cycle (Cori & Cori, 1929). Excess lactate produced through anaerobic respiration in peripheral tissues is transported to the liver and converted back to glucose via gluconeogenesis. During this process, bicarbonate is produced on a 1:1 basis as a by‐product of lactate metabolism, which helps to correct the acid–base imbalance (Heering et al., 1999; Katopodis et al., 2022). However, in sepsis, this lactate‐mediated bicarbonate generation may not be happening fast enough to compensate for the elevated lactate production. Studies using rat models of sepsis have shown that gluconeogenesis from lactate is significantly reduced in the livers of septic rats compared to healthy controls (Clemens et al., 1983; de Souza Galia et al., 2021). Potential reasons for impaired lactate‐driven gluconeogenesis in sepsis include reduced activity of essential enzymes involved in gluconeogenesis (i.e., pyruvate carboxylase, phosphoenolpyruvate carboxy kinase) (Deutschman et al., 1993; Wang et al., 2015). Impaired mitochondrial respiration decreases ATP availability for energy‐demanding gluconeogenesis (de Souza Galia et al., 2021; Rognstad, 1982). Therefore, the increase in PD along the pH‐bicarbonate axis in non‐survivors may reflect dysregulation of the acid–base compensatory system, particularly in the balance between lactate production and clearance.

Respiratory compensation is another essential mechanism that regulates the acid–base homeostasis; however, due to limited data availability, it was not evaluated in our study. Normally, a decrease in blood pH (e.g., metabolic acidosis) activates central and peripheral chemoreceptors, leading to an increase in the ventilation rate. Pulmonary expiration of carbon dioxide shifts the acid–base equilibrium (CO_2_ + H_2_O *⇌* H_2_CO_3_ *⇌* H^+^ + HCO_3_^−^) to the left, reducing the plasma concentration of hydrogen ions and thereby restoring blood pH towards physiological levels (Guyton & Hall, 2006). Sepsis can lead to respiratory failure, such as acute respiratory distress syndrome, and this may potentially impair respiratory regulation of the acid–base balance (Sheu et al., 2010). Future studies should incorporate respiratory parameters – including fraction of inspired oxygen (FiO_2_), partial pressure of oxygen (PaO_2_) and carbon dioxide (PaCO_2_) – into parenclitic network analysis to evaluate the role of the respiratory compensation in sepsis‐induced acid–base disturbances and its impact on patient outcomes.

Overall, the ability of PD(pH‐HCO_3_^−^) and PD(lactate‐pH) to predict 30‐day sepsis survival independent of other markers of sepsis severity support the critical roles of cellular metabolism and acid–base compensatory mechanisms in shaping the early course of sepsis. Furthermore, these findings highlight that parenchymal analysis not only predicts sepsis survival but also provides useful insights into sepsis pathophysiology and differences in physiological responses between survivors and non‐survivors.

### 4.3 Conventional (i.e., SOFA) versus network physiological approaches (i.e., PD and TE) for sepsis evaluation

One of our main goals was to assess whether network physiological techniques, such as parenclitic deviation, could offer a more effective evaluation of early sepsis outcomes compared to conventional approaches like SOFA. Our findings showed that both SOFA and PDs for pH‐HCO_3_^−^ and lactate‐pH axes were predictive of 30‐day survival but not 48‐h deterioration (Tables 4 and 5). Hence, we performed ROC curve analysis to compare the diagnostic performances between parenclitic network mapping and SOFA in predicting sepsis survival.

Our results show that the AUC for PDs across pH‐HCO_3_^−^ (0.723) and lactate‐pH (0.797) axes were higher compared to SOFA (0.708), indicating that PD had a better specificity and specificity at predicting 30‐day sepsis survival compared to SOFA. However, the difference was not statistically significant. We also plotted Kaplan–Meier graphs for PDs and SOFA, using the Youden Index from the ROC curve analysis as a cut‐off for classifying patients into predicted survivors and non‐survivor groups (Figure 3b). The Kaplan–Meier graphs showed that the difference in survival rates between patients above and below the cut‐off for PDs along pH‐HCO_3_^−^ (∆ = 31.5%) and lactate‐pH (∆ = 43.8%) axes was around two folds larger than the difference observed for SOFA (∆ = 18.7%). This finding suggests that the cut‐offs for PDs have a stronger ability to differentiate survivors and non‐survivors than SOFA, supporting the idea that network physiological approaches like parenclitic network mapping may perform better at evaluating sepsis survival than other linear techniques.

Earlier in the introduction, we mentioned that there are at least two different techniques for studying organ network interactions within individual patients. The first technique, known as dynamic network mapping (i.e., transfer entropy), analyzes time‐series data such as dynamical changes in vital sign recordings (heart rate, respiratory rate and oxygen saturation). The second method, called parenclitic network mapping (i.e., a static network mapping), computes cross‐sectional data like blood test results. Dynamic network mapping was previously investigated in sepsis by Morandotti et al. (2025). Building on this work, the authors investigated the application of static network mapping in predicting sepsis outcomes, using the same sepsis population included in the earlier work by Morandotti et al. (2025). We were interested in comparing the effectiveness of dynamic (transfer entropy) and static (parenclitic deviation) network mapping techniques in predicting sepsis outcomes. Hence, we compared our findings with the work by Morandotti et al., which looked at information transfer between the cardiorespiratory parameters – heart rate (HR), respiratory rate (RR), and blood oxygen saturation (SpO_2_) – using transfer entropy and evaluated whether it could predict early sepsis deterioration and survival (Morandotti et al., 2025). Similarly to our findings with PD, Morandotti et al. found that the transfer entropy (TE) values, representing the amount of information transferred between SpO2 → HR, HR → RR, and RR → HR, were significantly predictive of 30‐day sepsis survival, independent of other markers of sepsis severity (Figure S1). Consequently, we compared the diagnostic performance between TE and PD using results from the ROC curve analysis. We found that AUCs for TEs (0.635–0.724) were comparable to those of SOFA (0.708), while the AUCs for PDs (0.723–0.797) were generally higher than those for TEs (Table S4). This result suggests that PDs are better than TEs at predicting 30‐day sepsis survival, as they demonstrate higher specificity and sensitivity for distinguishing potential sepsis survivors from non‐survivors. However, unlike PD or SOFA, TEs were significantly predictive of 48‐h deterioration, independent of other factors impacting sepsis outcomes (Figure S2).

Collectively, this information offers several useful clinical insights. The fact that PD has a better predictive performance at classifying 30‐day sepsis survival implies that in resource‐limited settings where obtaining clean time‐series data is challenging, PD analysis using laboratory data can supplement SOFA to improve the identification of patients at risk of having an early sepsis death. On the other hand, the inability of the parenclitic analysis to predict 48‐h deterioration, unlike transfer entropy, indicates that a static network approach using point‐in‐time data could potentially overlook subtle physiological changes happening over time, which is otherwise detected in the dynamic network mapping.

Network physiological approaches, including dynamic network mapping studied by Morandotti et al. (2025) and static network mapping investigated in the present work, offered independent information to SOFA and performed better at predicting sepsis outcomes. This supports the limitations of the linear model of sepsis (i.e., SOFA) in evaluating sepsis and highlights the potential role of network physiological approaches in improving early detection of sepsis outcomes.

### 4.4 Future perspectives

Parenclitic deviation (PD) not only predicted early sepsis mortality but also provided valuable insight into individual patients' disease pathogenesis. Employing PD in ICU settings may improve understanding of distinct biological phenotypes within the sepsis population and potentially facilitate the development of personalized treatments based on physiological network map generated for each patient (Iglesias et al., 2020; Oyelade et al., 2023; Singer, 2019).

In this study, parenclitic network mapping was computed at a single time point the first day of ICU admission. However, in future studies, evaluating the variability of physiological variables and parenclitic networks across different time points may provide valuable insight into the temporal evolution of organ network connectivity and how these contribute to adaptive or maladaptive responses in sepsis.

Furthermore, both transfer entropy studied by Morandotti et al. (2025) and parenclitic deviation investigated in the present study offered independent information beyond SOFA for predicting sepsis outcomes in the same patient cohort. In future studies, incorporating additional measures of complexity (e.g., variability, fractal dynamics) and evaluating the unique perspectives into physiological network interactions captured by different techniques may further enhance our understanding of the complex sepsis pathophysiology (Buchan et al., 2012; Goldberger et al., 2002).

### 4.5 Limitations

Parenclitic network mapping is a promising approach for evaluating sepsis outcomes; however, several limitations need to be considered. The sample size of our study cohort (*n* = 162) was small due to the strict exclusion criteria requiring both clean time‐series and laboratory data. This limitation affects the statistical power of our results.

Our study was a retrospective cohort study investigating patient data from the MIMIC‐III database. The retrospective nature of the study introduces a risk of selection bias (Talari & Goyal, 2020). Additionally, the single‐centre nature of our patient data, collected from a single hospital in North America, resulted in a homogenous study population. This limitation restricts the generalisability of our findings to the broader sepsis population. To assess the applicability of parenclitic network mapping across a heterogenous sepsis population with diverse clinical and demographic backgrounds, findings from our study require validation using a larger, multi‐center cohort.

There were further limitations within our statistical analysis. For population‐level correlation analysis, we used parametric correlation analysis; in the upcoming studies, using other non‐parametric alternatives would be more generalisable, as it does not hold any assumptions about the underlying data distribution. Subsequently, for the multivariate Cox regression, although we have adjusted for the SOFA score, ventilation status, Elixhauser Comorbidity Index, age, gender, and ethnicity, other markers of sepsis severity, such as the use of medications (e.g., beta‐blockers) were not included (Chu et al., 2024). Future studies should investigate whether these additional factors influence the prognostic ability of PD.

Finally, the present work was a proof‐of‐concept study, evaluating the potential of parenclitic network mapping in predicting early sepsis outcomes. In future studies, we aim to follow the PROGRESS framework (Steyerberg et al., 2013). We hope to improve the clinical applicability of our findings by using large, good‐quality datasets, validating our findings in independent cohorts (as reference population) across different study regions, and developing more robust prognostic models that incorporate additional clinical markers (e.g., respiratory parameters and medication use) (Steyerberg et al., 2013).

## 5 CONCLUSION

Parenclitic deviation predicted 30‐day mortality in sepsis patients but not 48‐h deterioration, independent of other markers of sepsis severity. This method may offer useful insight into sepsis pathophysiology and different physiological responses between sepsis patients with various disease outcomes. Parenclitic network mapping analyses routine laboratory data and is a promising tool for early identification and treatment of at‐risk sepsis sub‐population in the clinical settings. Further research is required to validate our findings in a larger multi‐centred sepsis cohort.

## AUTHOR CONTRIBUTIONS

E.I., T.O., M.W., W.L., and A.R.M. conceived and designed research; E.I., M.W., J.L., and T.O. analyzed data; E.I. and A.R.M. interpreted results of experiments; E.I. prepared figures; E.I. drafted manuscript; T.O., J.L., and A.R.M. edited and revised manuscript; E.I., T.O., M.W., J.L., W.L., and A.R.M. approved final version of manuscript.

## CONFLICT OF INTEREST STATEMENT

No conflicts of interest, financial, or otherwise, are declared by the authors.

## FUNDING INFORMATION

This study was supported by UCL, with no external funding received.

## ETHICS STATEMENT

MIMIC‐III is publicly available to researchers under a data use agreement. The data has been deidentified according to HIPAA standards and the project was approved by the Institutional Review Boards of Beth Israel Deaconess Medical Center and MIT (IRB protocol nos. 2001P001699/14 and 0403000206). Individual patient consent was waived as the project did not affect clinical care and all protected health information was deidentified. The authors involved in data extraction completed mandatory online ethics training at MIT and were credentialed (ID 10304625).

## Supporting information


Figure S1.

Table S1.

## Data Availability

Data will be made available upon reasonable request.
