# Supplementary material for: Parenclitic network mapping predicts survival in critically ill patients with sepsis
Source: Physiol Rep. 2025 Jun 6;13(11):e70407. doi: 10.14814/phy2.70407 (PMC12141926; doi:10.14814/phy2.70407)

**Supplementary Figure S1:** Visualisation of parenclitic (PD) and transfer entropy (TE) networks for 30-day survival. The images of the TE network were adopted from Morandotti et al. (2025). For the PD networks (left column), the nodes represent the physiological variables, the edges show significant correlations (p≤ Bonferroni-adjusted p-value) between the two nodes, and the edge labels display the mean PD values for survivors and non-survivor groups along the significantly correlated variable pair axes. For the TE networks (right column), the nodes represent cardiorespiratory parameters, the edges illustrate the causal relationship between the parameters, and the edge labels show the average amount of information transferred (in bits) for the sepsis population with different survival outcomes. For both PD and TE networks, the edges that significantly predict 30-day survival independent of other markers of sepsis severity (p<0.05) are highlighted in red. The edges highlighted for PD predicted survival independent of SOFA and ventilation status. The edges highlighted for TE predicted survival independent of additional covariates including age, beta blocker use, Elixhauser comorbidity index, SOFA, and ventilation status. Abbreviations: haem=haemoglobin, pH = arterial pH, HCO3 = bicarbonate, inr = international normalised ratio, ALT = alanine transaminase, wbc = white blood cell, HR = heart rate, RR = respiratory rate, SpO_2_ = oxygen saturation.


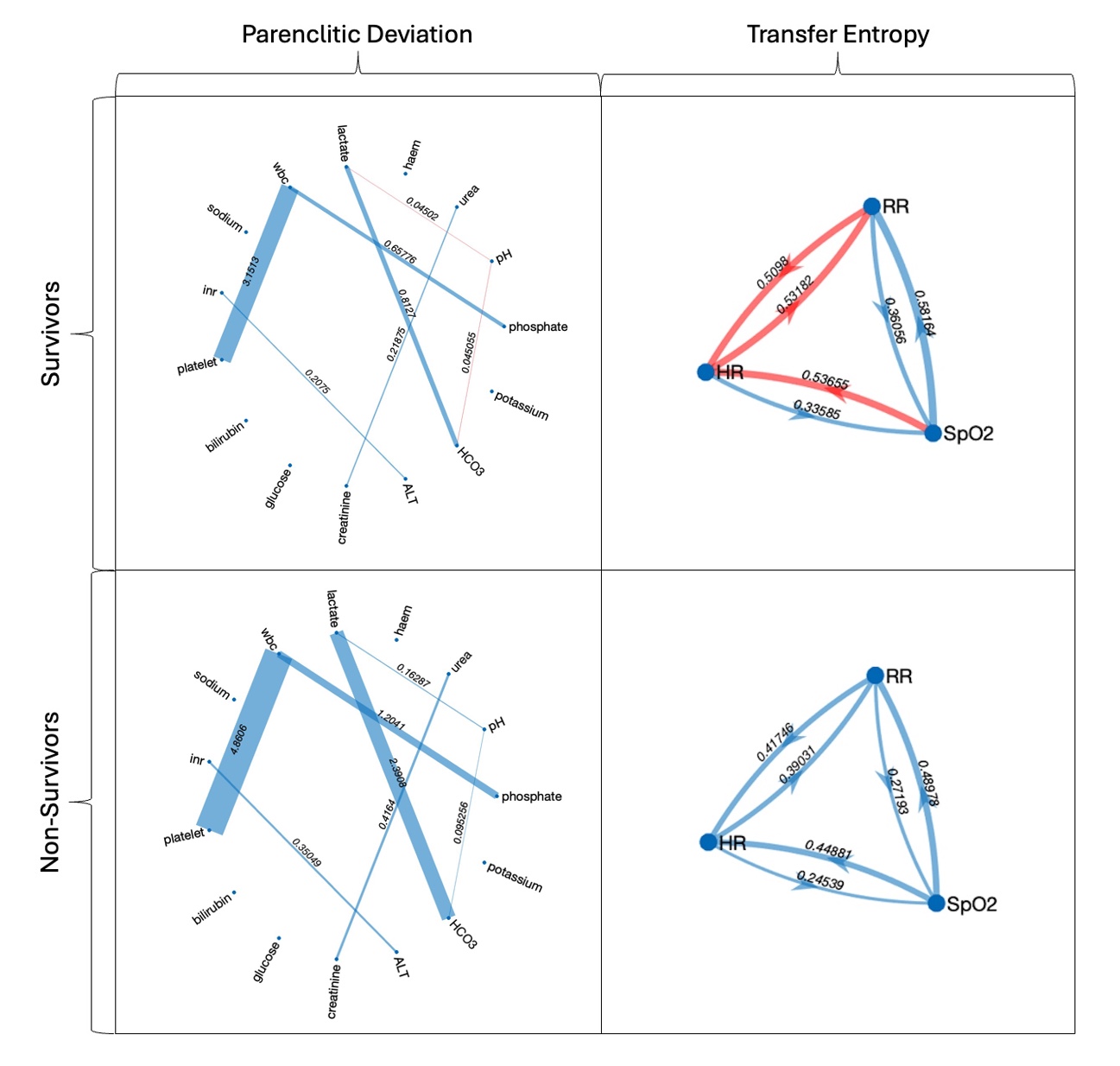


**Supplementary Figure S2:** Visualisation of parenclitic (PD) and transfer entropy (TE) networks for 48-hour deterioration. The images of the TE network were adopted from Morandotti et al. (2025). For the PD networks (left column), the nodes represent the physiological variables, the edges show significant correlations (p≤ Bonferroni-adjusted p-value) between the two nodes, and the edge labels display the mean PD values for no deterioration and deterioration groups along the significantly correlated variable pair axes. For the TE networks (right column), the nodes represent cardiorespiratory parameters, the edges illustrate the causal relationship between the parameters, and the edge labels show the average amount of information transferred (in bits) for the sepsis population with different deterioration outcomes. For both PD and TE networks, the edges that significantly predict 48-hour deterioration independent of other markers of sepsis severity (p<0.05) are highlighted in red. None of the edges for PD predicted 48-hour deterioration. The edges highlighted for TE predicted deterioration independent of additional covariates including age, beta blocker use, Elixhauser comorbidity index, SOFA, and ventilation status. Abbreviations: haem=haemoglobin, pH = arterial pH, HCO3 = bicarbonate, inr = international normalised ratio, ALT = alanine transaminase, wbc = white blood cell, HR = heart rate, RR = respiratory rate, SpO_2_ = oxygen saturation.


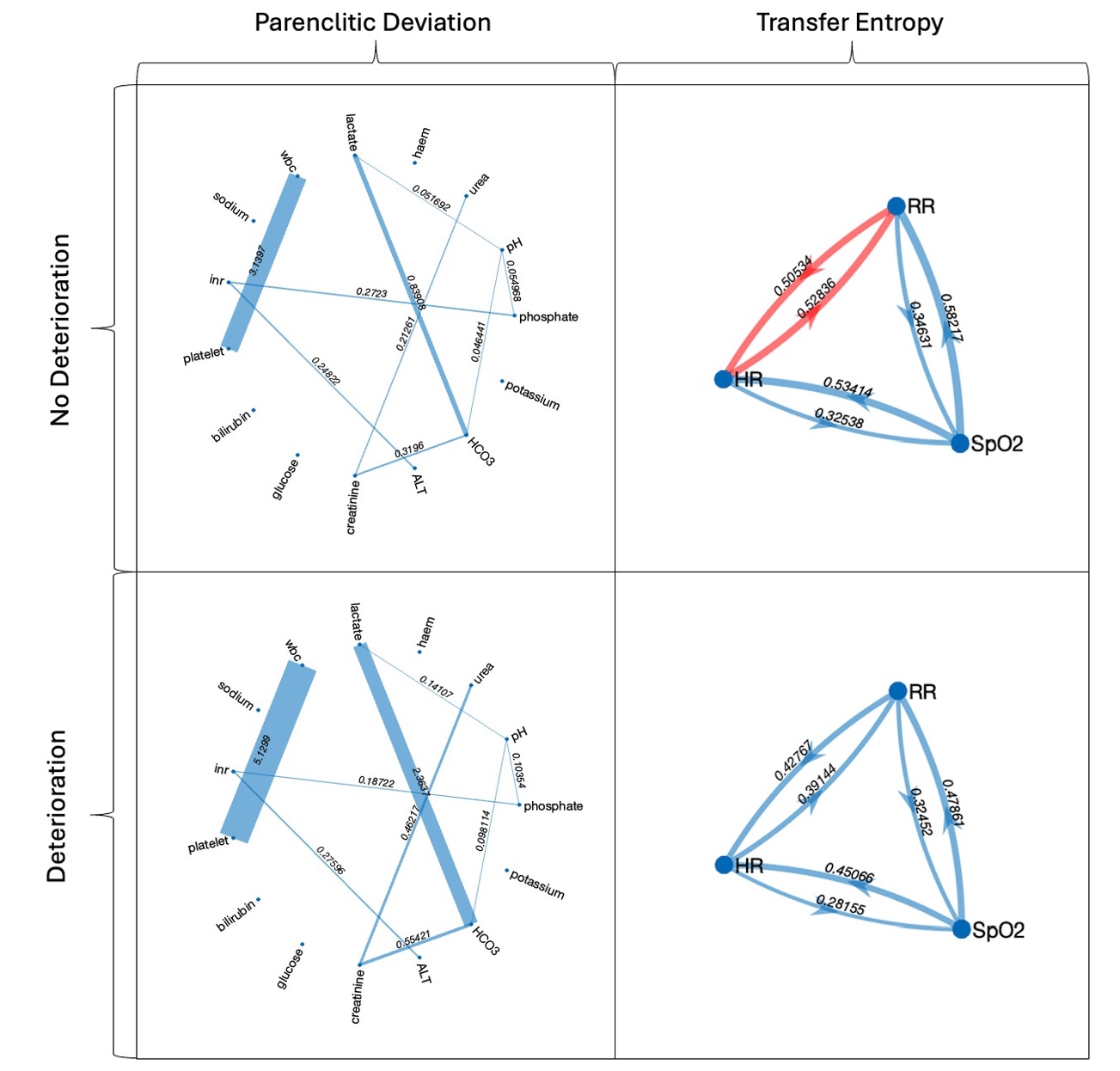

Supplement: Supplementary file 1 — Figure S1. [file PHY2-13-e70407-s002.docx]
