# Supplementary material for: Parenclitic network mapping predicts survival in critically ill patients with sepsis
Source: Physiol Rep. 2025 Jun 6;13(11):e70407. doi: 10.14814/phy2.70407 (PMC12141926; doi:10.14814/phy2.70407)
Supplement: Supplementary file 2 — Table S1. [file PHY2-13-e70407-s001.docx]

**Supplementary Table S1:** Results from multivariate Cox regression evaluating whether parenclitic deviations (PD) that significantly predicted 30-day mortality independent of SOFA and ventilation status remained predictive of survival after adjusting for additional factors including comorbidity, age, gender, and ethnicity. All PD values were normalised with Z-transformation prior to analysis. Significant predictors of 30-day mortality (p < 0.05) are highlighted in bold. A dash (-) indicates results that could not be computed due to insufficient sample size within certain ethnic groups.

|  |  |  |  | **95% CI (Hazard Ratio)** | |  |
| --- | --- | --- | --- | --- | --- | --- |
| **Covariates** | $\boldsymbol{\beta}$ | **SE** | **Hazard Ratio** | **Lower** | **Upper** | **P-value** |
| *Multivariate Cox regression analysis for PD (pH - HCO_3_^-^)* | | | | | | |
| PD (pH - HCO_3_^-^) | 0.944 | 0.233 | 2.570 | 1.628 | 4.056 | **<0.001** |
| Arterial_pH | -0.015 | 0.293 | 0.985 | 0.554 | 1.751 | 0.959 |
| HCO_3_^-^ | -0.354 | 0.252 | 0.702 | 0.428 | 1.150 | 0.160 |
| SOFA | 0.095 | 0.070 | 1.100 | 0.960 | 1.261 | 0.170 |
| Ventilation Status | 1.215 | 0.479 | 3.372 | 1.318 | 8.624 | **0.011** |
| Elixhauser Score | 0.016 | 0.039 | 1.017 | 0.941 | 1.098 | 0.677 |
| Age | 0.060 | 0.017 | 1.062 | 1.028 | 1.097 | **<0.001** |
| Gender (Male) | Reference Group | | | | | |
| Gender (Female) | -0.099 | 0.509 | 0.906 | 0.334 | 2.455 | 0.846 |
| Ethnicity (White) | Reference Group | | | | | |
| Ethnicity (Asian) | - | - | - | - | - | - |
| Ethnicity (Black) | ﻿-1.195 | 0.829 | 0.303 | 0.060 | 1.536 | 0.149 |
| Ethnicity (Hispanic) | 2.387 | 0.849 | 10.885 | 2.060 | 57.510 | **0.005** |
| Ethnicity (Others) | - | - | - | - | - | - |
| Ethnicity (Unknown) | 0.132 | 0.580 | 1.141 | 0.366 | 3.555 | 0.821 |
| *Multivariate Cox regression analysis for PD (lactate - pH^-^)* | | | | | | |
| PD (lactate - pH) | 1.400 | 0.522 | 4.055 | 1.459 | 11.273 | **0.007** |
| Lactate | 0.040 | 0.365 | 1.041 | 0.509 | 2.128 | 0.913 |
| Arterial_pH | 0.006 | 0.429 | 1.006 | 0.434 | 2.330 | 0.989 |
| SOFA | 0.033 | 0.096 | 1.034 | 0.857 | 1.247 | 0.729 |
| Ventilation Status | 0.860 | 0.563 | 2.363 | 0.784 | 7.122 | 0.126 |
| Elixhauser Score | 0.014 | 0.040 | 1.014 | 0.936 | 1.097 | 0.736 |
| Age | 0.052 | 0.020 | 1.054 | 1.013 | 1.097 | **0.010** |
| Gender (Male) | Reference Group | | | | | |
| Gender (Female) | -0.024 | 0.574 | 0.977 | 0.317 | 3.011 | 0.967 |
| Ethnicity (White) | Reference Group | | | | | |
| Ethnicity (Asian) | - | - | - | - | - | - |
| Ethnicity (Black) | -1.172 | 0.948 | 0.310 | 0.048 | 1.985 | 0.216 |
| Ethnicity (Hispanic) | - | - | - | - | - | - |
| Ethnicity (Others) | - | - | - | - | - | - |
| Ethnicity (Unknown) | 0.403 | 0.689 | 1.497 | 0.388 | 5.775 | 0.558 |

**Supplementary Table S2:** Results from ROC Curve Analysis of parenclitic deviations significantly predictive of 30-day mortality (PDs for pH-HCO_3_^-^ and lactate-pH axes) and SOFA (p<0.05). To allow comparison of ROC curves between PDs and SOFA using Delong’s test, areas under the curve (AUCs) were calculated amongst patients (N = 72) with complete data available for SOFA, PD (pH - HCO3-) and PD (lactate – pH).

|  |  | **95% CI for AUC** | |  |
| --- | --- | --- | --- | --- |
| **Variables** | **AUC** | **Lower** | **Upper** | **P value** |
| PD (pH - HCO_3_^-^) | 0.784 | 0.652 | 0.915 | **0.000** |
| PD (lactate - pH) | 0.786 | 0.651 | 0.921 | **0.000** |
| SOFA | 0.734 | 0.590 | 0.879 | **0.001** |

**Supplementary Table S3:** Pairwise comparison of ROC curves between parenclitic deviations significantly predictive of 30-day mortality (PDs for pH-HCO_3_^-^ and lactate-pH axes) and SOFA (p<0.05) using Delong’s test. The differences between AUCs ($\Delta$AUC) were calculated amongst patients (N = 72) with complete data available for SOFA, PD (pH - HCO3-) and PD (lactate - pH). Abbreviation: $\Delta$AUC, Difference between Areas under the Curve; CI, Confidence Interval.

|  |  | **95% CI for AUC** | |  |  |
| --- | --- | --- | --- | --- | --- |
| **Compared Variable Pairs** | $\boldsymbol{\Delta}$**AUC** | **Lower** | **Upper** | **Z statistic** | **P value** |
| SOFA and PD (pH - HCO_3_^-^) | 0.049 | -0.077 | 0.175 | 0.764 | 0.445 |
| SOFA and PD (lactate - pH) | 0.052 | -0.124 | 0.227 | 0.577 | 0.577 |
| PD (pH - HCO_3_^-^) and PD (lactate - pH) | 0.003 | -0.156 | 0.161 | 0.031 | 0.976 |

**Supplementary Table S4:** Comparison of the results from the ROC curve analysis for parenclitic deviations and transfer entropies that significantly predict 30-day mortality independently of other markers of sepsis severity (p<0.05) and SOFA. All the results for transfer entropy were adopted from Morandotti et al. (2025). Abbreviations: AUC = Area Under the Curve, CI = confidence interval, pH = arterial pH, HCO3 = bicarbonate, HR = heart rate, RR = respiratory rate, SpO_2_ = oxygen saturation.

|  |  | **95% CI for AUC** | |  |  |
| --- | --- | --- | --- | --- | --- |
| **Parameters** | **AUC** | **Lower** | **Upper** | **P value** | **Youden index** |
| TE (HR$\to$RR) | 0.724 | 0.622 | 0.827 | **0.000** | 0.526908013 |
| TE (RR$\to$HR) | 0.635 | 0.518 | 0.751 | **0.016** | 0.468135181 |
| TE (SpO_2_$\to$HR) | 0.658 | 0.554 | 0.761 | **0.005** | 0.560797664 |
| PD (pH - HCO3) | 0.723 | 0.607 | 0.839 | **0.001** | 0.050571020 |
| PD (lactate - pH) | 0.797 | 0.667 | 0.927 | **0.000** | 0.071192436 |
| SOFA | 0.708 | 0.602 | 0.814 | **0.000** | 4.5 |
